# Decluttering

**DOI:** 10.37825/2239-9747.1002

**Published:** 2020-10-01

**Authors:** C Ciacci, O Piazza

**Affiliations:** 1 Gastroenterology, Department of Medicine, Surgery, Dentistry, Scuola Medica Salernitana, University of Salerno, Italy; 2 Intensive Care, Department of Medicine, Surgery, Dentistry, Scuola Medica Salernitana, University of Salerno, Italy

**Keywords:** COVID-19, lockdown, telemedicine

## COMMENTARY

From Wikipedia: “.. *in literature, confessional writing is a first-person style that is often presented as an ongoing diary or letters, distinguished by revelations of a person’s deeper or darker motivations*.”

Here we are, academics, researchers, physicians expressing our point of view on the COVID-19 pandemic in a confessional style.

We have heard and read many personal experiences from colleagues on the battlefront. In all of them, we could feel the mix of fear, discouragement but also pride for being there, on duty.

We live and work not far from the front line, but we are not either in the midst of the storm. Since the end of February 2020, we frantically tried to get ready for the “tsunami” we dreaded to arrive, avidly collecting scientific information to prepare a strategy, requiring mechanical ventilators, drugs and intensive care equipment that never arrived, creating a modern and efficient isolation ICU room in an ancient and fascinating building. And, in the meanwhile, we had to put on hold routine follow-up visits, and clinical trials. Everything we were used to was stopped or was on a sinister, unnatural hold. Hospitals admit only emergencies even today, and we are working in a never experienced low pressure. Due to the lack of protective masks and to respect social distancing in a crowded hospital, the personnel started smart working and using telemedicine from home. So, did we, at home every weird afternoon.

Meanwhile, meetings and events were cancelled. No travelling for us: that means no last-second trains, no flights, no junk food at airports, while waiting for a late plane, no nights in unremarkable hotels, no presentations to prepare at the very last minute. Like many others, we are living in a suspended time, made up of long quiet hours at home, interrupted by friendly and compassionate video calls with patients and students.

In about 60 days of lockdown, we had the time to study with fresh and juvenile attention the Sars-COV-2 infection, to finish some forgotten papers waiting hidden in our computers folders. Doing so, we cleaned the desktop of our computers and we even correctly classified our files, in order to find them in the future. We had the time to renew and improve our presentations for the e-learning classes, to practice more and more efficient telemedicine. We admit that the quantity and the quality of our work as teachers and even as physicians have improved.

Like most people of the world, and as doctors, we are suffering from the burden of a disease that caused so many deaths, so much sorrow, too many inequalities. Sure, we miss our family and friends. But we do not miss the rest, since in the protective environment of our super sanitized home and offices, we are discovering the pleasure of decluttering, a new, or better, a forgotten dimension of living.

The virus changed our lives, and we share the universal feeling of uncertainty for the future. Nevertheless, we confess in a whisper: we feel lucky and are enjoying the present suspended time. The lockdown experience locked us down physically but not spiritually. It has been, at least for some of us, a good alibi for taking a break. It was not our fault, and we cannot feel bad for having missed so many meetings, while comfortably working at home. For a scientist working means thinking and reflecting and rethinking, too. You can say we are selfish but, please, believe us, we feel horrible for our colleagues who died on the battlefront without perspective and adequate protections. In the effort of being honest, we must confess that, but despite so many limitations, we learned a lot about the unrevealed, meaningless academic overload, and our working and personal life is easier now than before the COVID-19 breakdown.

In a way, we reencountered our young self, and we found the lost time to enjoy small talks. We decluttered our closets and life. We are sure that we are not the only ones. However, the ‘protected’ condition will not and should not last. We will take with constructive spirit the only advantage of the immense tragedy of the pandemic. Hopefully, we will make sure we will not forget to save some time for us and to learn how to say “no, thanks” to many unnecessary carrousels.

